# The Structure and Function of the Glycocalyx and Its Connection With Blood-Brain Barrier

**DOI:** 10.3389/fncel.2021.739699

**Published:** 2021-10-07

**Authors:** Jing Jin, Fuquan Fang, Wei Gao, Hanjian Chen, Jiali Wen, Xuehua Wen, Junfa Chen

**Affiliations:** ^1^Zhejiang Center for Clinical Laboratory, Zhejiang Provincial People’s Hospital, Affiliated People’s Hospital, Hangzhou Medical College, Hangzhou, China; ^2^Department of Anesthesiology, The First Affiliated Hospital, Zhejiang University School of Medicine, Hangzhou, China; ^3^The First Affiliated Hospital of Wenzhou Medical University, Wenzhou, China; ^4^Department of Radiology, Zhejiang Provincial People’s Hospital, Affiliated People’s Hospital, Hangzhou Medical College, Hangzhou, China

**Keywords:** glycocalyx, blood-brain barrier, neurovascular unit, neurological function, neurological diseases

## Abstract

The vascular endothelial glycocalyx is a dense, bush-like structure that is synthesized and secreted by endothelial cells and evenly distributed on the surface of vascular endothelial cells. The blood-brain barrier (BBB) is mainly composed of pericytes endothelial cells, glycocalyx, basement membranes, and astrocytes. The glycocalyx in the BBB plays an indispensable role in many important physiological functions, including vascular permeability, inflammation, blood coagulation, and the synthesis of nitric oxide. Damage to the fragile glycocalyx can lead to increased permeability of the BBB, tissue edema, glial cell activation, up-regulation of inflammatory chemokines expression, and ultimately brain tissue damage, leading to increased mortality. This article reviews the important role that glycocalyx plays in the physiological function of the BBB. The review may provide some basis for the research direction of neurological diseases and a theoretical basis for the diagnosis and treatment of neurological diseases.

## Introduction

The surface of the vascular endothelium is covered with a layer of villiform substance, which is called the glycocalyx. It is synthesized by vascular endothelial cells and extends to vascular lumen and surface. In 1966, Rambourg et al. (1966) used methylamphetamine labeled with Ag to observe a layer of proteoglycan (PG) protein polymers on the surface of endothelial cells of mice for the first time. With the development of modern methods of fixation and rapid-freeze techniques as well as a variety of confocal microscopy, there have been more in-depth studies on the structure and functions of the glycocalyx (Ebong et al., 2011). The glycocalyx on endothelial cells is a kind of PG polymer. It mainly includes PGs and glycosaminoglycan chains (GAGs). The core protein of PG mainly consists of members of syndecan and glypican families. GAGs, including heparan sulfate (HS), chondroitin sulfate (CS), and hyaluronan (HA), are the most abundant components of the glycocalyx (Salmon and Satchell, 2012; Alphonsus and Rodseth, 2014; Mende et al., 2016). Glycocalyx extends from the membrane of endothelial cells to vascular lumen, prevents leukocytes and platelets from contacting with endothelial cells, and plays a key role in maintaining the stability of the internal environment (Salmon and Satchell, 2012; Ushiyama et al., 2016). Research has proved that glycocalyx can maintain the stability of many physiological functions, such as maintaining the permeable barrier of microcirculation, preventing trigger inflammation and coagulation response, and conducting the shear force of blood flow (Iba and Levy, 2019; Nikmanesh et al., 2019; Zuurbier, 2019). It can also protect the functions of vital organs including the brain, heart, lungs, and kidneys (Becker et al., 2010b; van Golen et al., 2014; Brettner et al., 2017; Rabelink et al., 2017; Zhu et al., 2017).

The BBB prevents sensitive neurons from being attacked by toxic metabolites and exogenous materials in the circulation. Therefore, stable and intact BBB is crucial for maintaining normal physiological functions of the brain. The cerebrovascular dysfunction, such as destruction of the BBB, endothelium dysfunction, or capillary degeneration, is also related to the pathogenesis and progression of many nervous system diseases, including neuroinflammation, cognitive decline related to aging, multiple sclerosis, brain tumor, and epilepsy (Zenaro et al., 2017; Abdullahi et al., 2018; Abrahamson and Ikonomovic, 2020). With the development of the confocal technique and photon fluorescence imaging technique, the microstructure of the BBB has gradually become clear to researchers. The unique system structure mainly consists of pericytes, endothelial cells, glycocalyx of endothelial cells, basement membrane, and astrocyte cells (Kutuzov et al., 2018; Santa-Maria et al., 2021).

After the glycocalyx in the endothelium of the BBB is impaired, a series of pathophysiological changes related to the microcirculation occurs. If the glycocalyx is degraded, the permeability of the BBB increases, leading to neuroedema. The number of leukocyte and platelets binding with the exposed surface receptors of endothelial cells increases, causing inflammation, a blood clotting response, cerebral microcirculation ischemia, and damage to the nervous tissue (Kutuzov et al., 2018; Zhao et al., 2021). Currently, there are few overviews of the glycocalyx and cerebrovascular microcirculation. In this review, we discuss the structure and physiological functions of endothelial glycocalyx and the progress of related research on endothelial glycocalyx and cerebral vessels in detail and provide some clues for subsequent research and disease treatment.

### The Structure of the Glycocalyx

The endothelial glycocalyx is a layer of dense and uneven grass-like substance covering the surface of vascular endothelial cells (Fang et al., 2021). The endothelial glycocalyx is a PG polymer synthesized and secreted by endothelial cells. Through the skeleton consisting of PG and glycoproteins (GLYs), it binds with endothelial cells. In this net structure, soluble factors from plasma and endothelial cells are bound and attached. This grass-like structure maintains the dynamic balance under physiological conditions. The main core PG proteins are members of the syndecan and glypican families. These core proteins firmly bind with the cell membrane and pass the membrane-spanning domain (syndecans) or a glycosylphosphatidylinositol anchor (glypicans) (Kabedev and Lobaskin, 2018; Purcell and Godula, 2019). The syndecan family comprises 5 members: syndecan-1, syndecan-2, syndecan-3, and syndecan-4. Among these members of the syndecan family, syndecan-1 expressed by vascular endothelial cells can bind HS, CS, and keratan sulfate. Syndecan is closely related to the shear force of blood flow (Koo et al., 2013). Members of the glypican family include glypican-1, glypican-2, glypican-3, glypican-4, glypican-5, and glypican-6. Glpyican-1 is the only member of the glypican family expressed on endothelial cells. The branch linkage includes HS (Tarbell, 2010).

The side chain of GAGs binds with the main part of core protein or CD44 receptors on the surface of endothelial cells. There are 5 types of GAGs, namely, HS, CS, dermatan sulfate, keratan sulfate, and HA (or hyaluronic acid). HS, CS, and dermatan sulfate with negative charges bind the core protein through covalent binding. HS is the most abundant components of GAG side chains, comprising 50–90% of these chains (Pries et al., 2000). The next most abundant components are CS and dermatan sulfate, whose content is approximately one-quarter of that of HS (Rapraeger et al., 1985). The details of keratan sulfate are currently unknown. In contrast to the four abovementioned GAGs, non-sulfated HA, which has no charge, does not bind the core protein, but covalently binds the cell membrane through CD44 receptors (Nandi et al., 2000). GAG chains with negative charges can bind plasma proteins and positively charged ions through the electric charge effect (Van den Berg et al., 2006; Reitsma et al., 2007).

**FIGURE 1:**
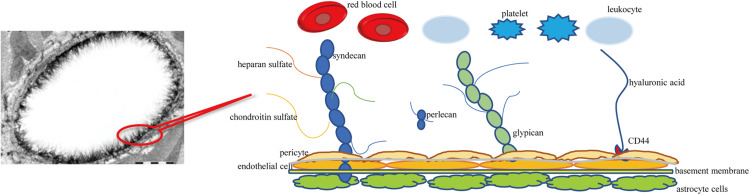
Schematic diagram of cerebral vascular endothelial glycocalyx. Syndecan and glypican are membrane-bound proteoglycans-core skeletons, to which chondroitin sulfate and heparan sulfate are connected. Syndecans are transmembrane proteoglycans. Glypicans attach to the cell surface through glycosylphosphatidylinositol anchors. Hyaluronic acid is connected to the endothelial cell membrane through the CD44 receptor.

Similar to PGs, GLYs are skeleton proteins of the glycocalyx that link the glycocalyx and endothelial cells. GLYs are adhesion molecules on the surface of endothelial cells. They mainly consist of members of the selectin family, the integrin family, and the immunoglobulin superfamily. The selectin family members that are expressed on the surface of endothelial cells mainly include E-selectin and P-selectin. They participate in the adhesion of leukocytes and endothelial cells (Sperandio, 2006). The main function of the integrin family on the surface of endothelial cells is mediating the adhesion of endothelial cells and platelets and the linkage of extracellular matrix, such as lantinin, fibronectin, and collagen (Bombeli et al., 1998; Ruegg and Mariotti, 2003). The immunoglobulin superfamily of glycocalyx includes the cytoplasmic domain, transmembrane domain, and intracellular domain. The main molecules include intercellular adhesion molecules 1 and 2 (ICAM-1 and –2), vascular cell adhesion molecule 1 (VCAM-1), and platelet/endothelial cell adhesion molecule 1 (PECAM-1) (Reitsma et al., 2007). It has been observed under an electron microscope that the thickness of the glycocalyx of the vascular endothelium is 0.1–11 μm (Becker et al., 2010a). A schematic diagram of cerebral vascular endothelial glycocalyx is shown in Figure 1.

### The Role of Glycocalyx in Permeability

**FIGURE 2:**
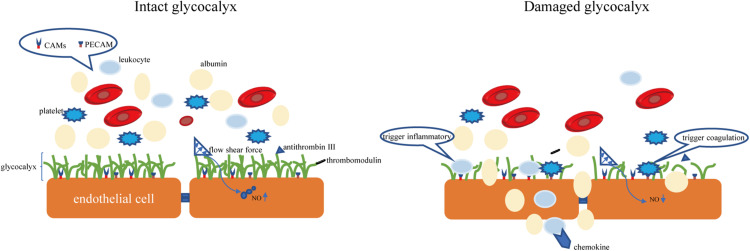
The intact glycocalyx can maintain the permeability of the blood-brain barrier. Damage to the glycocalyx will increase the permeability of the blood-brain barrier (for example, albumin exudation). The CAMs and PECAM hidden in the glycocalyx are exposed due to the shedding of the glycocalyx, causing the aggregation of leukocytes and platelets. CAMs, endothelial cell adhesion molecules. PECAM, platelet/endothelial cell adhesion molecule.

The endothelial glycocalyx is an important gatekeeper of vascular permeability. Damage to the glycocalyx increases the transport of water, proteins, and other molecules from the plasma to outside of blood vessels (Butler et al., 2020). The endothelial glycocalyx can restrict certain molecules from passing through the endothelial cell membrane, as confirmed by injecting of fluorescently labeled dextran into rat mesenteric arteries (van Haaren et al., 2003). It was observed that the in rat myocardial capillaries, the glycocalyx is degraded by enzymes, and the subsequent hyperosmolarity leads to myocardial edema (Araibi et al., 2020). Degradation of 42% of the endothelial glycocalyx in the isolated rat abdominal aorta by hyaluronidase (HAase) facilitates water and low-density lipoprotein transport across the vessel wall, suggesting that the endothelial glycocalyx is a transport barrier (Kang et al., 2021). Not only does the molecular sieve effect of the glycocalyx structure determine the permeability of blood vessels, but the negatively charged nature of glycocalyx also makes blood vessels act as a charge barrier. Heparan sulfate and chondroitin sulfate in glycosaminoglycan side chain components are negatively charged, so the glycocalyx facing the plasma is also negatively charged. Studies have found that neutralizing the negative charge of the glycocalyx by myeloperoxidase can induce permeability and increase vascular permeability (Kolarova et al., 2021). According to the traditional Starling model, two opposite forces passing through the endothelial cell layer maintain fluid distribution balance, which is determined by four factors: capillary pressure, tissue fluid hydrostatic pressure, plasma colloid osmotic pressure, and tissue fluid colloid osmotic pressure (Starling, 1896). In recent years, the discovery of microvascular barrier functions has questioned this notion, suggesting that the structural net consisting of the endothelial glycocalyx binds with the endothelial cell membrane of blood vessels and forms the endothelial surface layer, which bears the blood vessel barrier. The resulting osmotic pressure of the transendothelial PG protein colloid is the main determining factor of the internal and external flow of fluid in capillaries (Michel, 1997). A schematic diagram of the physiological functions of glycocalyx is shown in Figure 2.

### The Role of Glycocalyx in Inflammation

The vascular response is the central part of the inflammatory response. Lipowsky et al. (2011) observed that, in a mouse model of inflammation, after the vascular endothelial glycocalyx structure is destroyed, vascular endothelial cell intercellular adhesion molecule-1 (ICAM-1) and vascular cell adhesion molecule-1 (VCAM-1) make it easier for leukocytes in the blood circulation to adhere to the vascular endothelial cells, which in turn cause a series of inflammation and pathological changes (Mulivor and Lipowsky, 2004; Devaraj et al., 2009; Mulivor and Lipowsky, 2009; Schmidt et al., 2012; Lipowsky and Lescanic, 2013). Therefore, glycocalyx shedding is the response of vascular endothelial cells to inflammatory mediators. In an inflammatory state, the glycocalyx of vascular endothelial cells falls off, but it also plays an important role in regulating the occurrence and development of inflammation. HS is the main component of the vascular endothelial glycocalyx and exists on the surface and matrix of cerebrovascular cells (Bernfield et al., 1999). A series of *in vitro* cell experiments confirmed that HS is a potential ligand of P and L-selectin, which binds to pro-inflammatory chemokines and promotes the transmembrane transport of chemokines (Hoogewerf et al., 1997; Koenig et al., 1998). Vascular endothelial HS participates in and regulates multiple stages of an inflammatory response, but its exact role in the process of inflammatory response is not fully understood.

### The Role of the Glycocalyx in the Anticoagulant Process

Glycocalyx’s dense and bush-like structure can hide coagulation-related molecules. Under physiological conditions, direct contact between endothelial cells and blood cells can be avoided, thereby avoiding thrombosis. In addition, glycocalyx can also achieve anticoagulant effects by interacting with antithrombin III, thrombomodulin, and tissue factor pathway inhibitor (TFPI). The main mechanisms of actions include (Bell et al., 2017; Lupu et al., 2020): (1) PECAM is exposed by the shed glycocalyx; (2) Antithrombin III binds to HS in the glycocalyx to enhance its anticoagulant effect; (3) Thrombomodulin can bind to CS to convert thrombin into the protein C activator of the pathway, thereby forming the anticoagulation pathway; (4) TFPI is an effective inhibitor of FVIIa and FXa in the coagulation pathway, and the anticoagulation effect is achieved mainly through the interaction of TFPI and glycocalyx (Kozar and Pati, 2015).

### The Glycocalyx as a Signal Sensor

The glycocalyx can sense changes in blood flow shear force and transmit it to endothelial cells, which induces corresponding morphological and functional responses, such as the release of endogenous vasoactive substances and nitric oxide (NO) and cytoskeleton changes (Lyu et al., 2020). In the rat blood vessel model, the amount of NO produced by blood vessels was detected after HS enzymatically degraded under changes of blood flow shear force. Researchers have found that the production of nitric oxide is significantly reduced (Yen et al., 2015). However, not all components of the glycocalyx can mediate shear-induced NO release. Anne Marie W Bartosch et al. (2017) used atomic force microscopy (AFM) to selectively apply forces onto glycocalyx components, including PGs and GAGs, to observe how each component individually promotes force-induced NO production. They concluded that HS and the glypican-1, not syndecan-1, CD44, and HA, are the main mechanical sensors for shear-induced NO production (Bartosch et al., 2017). According to the report of Eno E Ebong, core protein syndecan-1 of HS mediates flow-induced endothelial cells elongation and alignment because SDC1 is linked to the cytoskeleton which impacts cell shape (Ebong et al., 2014). Kang et al. (2021) found that 24-h shear exposure increased the average maximum infiltration distance of low-density lipoprotein and enhanced endothelial cells apoptosis and that both of these effects were inhibited by HAase, indicating that the glycocalyx of endothelial cells can also serve as shearing mechanical sensors regulate endothelial cell apoptosis, thereby affecting leaky connections and regulating low-density lipoprotein transport.

### The Effect of the Endothelial Glycocalyx in Cerebrovascular Micro-Homeostasis

The BBB is a unique structure that is mainly composed of pericytes, endothelial cells, the glycocalyx, basement membranes, and astrocytes (Kutuzov et al., 2018). Glycocalyx plays an irreplaceable role in maintaining the barrier function of cerebral blood vessels. Through EB and IgG extravasation assays, Zhu et al. (2018) found that in the group with integral glycocalyx structure, EB and IgG did not leak into the hippocampus. However, upon treatment with heparanase (HPSE), leakage was obvious (Zhu et al., 2018). The glycocalyx can prevent some molecules from passing through the BBB. Kutuzov et al. (2018) used a two-photon microscopy to observe the transport of four different sizes of molecules, i.e., fluorescein sodium (376 Da), Alexa Fluor (643 Da), 40-kDa dextran, and 150-kDa dextran from blood to the brain tissue in the cortical capillaries of anesthetized mice. Fluorescein and Alexa penetrate almost the entire glycocalyx structure layer, while the penetration rate of dextran is less than 60% of the volume. This indicates that glycocalyx can block large molecules in the BBB very well, but the ability to prevent small molecules from infiltrating is limited (Kutuzov et al., 2018). In the rat cardiac arrest/cardiopulmonary resuscitation model, the degree of glycocalyx destruction caused by HAase treatment was related to the high BBB permeability and aggravation of cerebral edema after circulation recovery and perfusion, as well as the decrease in survival rate at day 7 and poor nervous system-related prognosis (Zhu et al., 2018). The mechanisms by which the glycocalyx maintains the permeability of the BBB mainly include the following. First, the dense bush-like structure can play a physical isolation effect (Kutuzov et al., 2018). Second, HS and CS in the side chain of GAGs carry negative charges. Therefore, glycocalyx can prevent negatively charged molecules such as albumin from passing through the BBB due to charge repulsion (Deen et al., 2001). And third, after damage to the endothelial glycocalyx, the levels of inflammatory molecules and matrix metalloproteinases (MMPs) increase, resulting in disruption of the close interactions that form the BBB and further increasing vascular permeability.

In addition to regulating the permeability of the blood-brain barrier (BBB), glycocalyx is also involved in cerebrovascular coagulation and neuroinflammatory processes (Lupu et al., 2020). Delayed cerebral ischemia is a common complication of aneurysmal subarachnoid hemorrhage, but the specific mechanism is not clear. Bell et al. (2017) study on patients with aneurysmal subarachnoid hemorrhage found that in patients with delayed cerebral ischemia, specific markers of glycocalyx damage, including SDC-1, were significantly elevated and that this elevation of syndecan-1 expression was related to vascular adhesion protein-1 in the plasma and endothelial cell adhesion molecules (CAMs) in the cerebrospinal fluid. This indicates that the breakdown of cerebrovascular glycocalyx integrity may be related to ischemic brain diseases (Bell et al., 2017). Moreover, the endothelial adhesion molecules ICAM-1 and VCAM-1 within the glycocalyx are exposed after glycocalyx degradation (Simard et al., 2012). This adhesion molecules are known as the central mediators of leukocyte adhesion to and transmigration across BBB (Schnoor et al., 2015). Upregulation of proinflammatory cytokines as a response to leakage of leucocytes further contributes to the subsequent increased neuronal excitability (Rana and Musto, 2018). Kim et al. found that after glycocalyx degradation, ICAM-1 and NF-kB not only increase leukocyte adhesion, but also up-regulate the expression of iNOS and COX-2 (Kim et al., 2013). Inflammatory factors such as TNF-α and oxygen free radicals increase the production of MMPs, which in turn damage brain tissue. The function, shedding enzyme and strategies of glycocalyx protection are summarized in Table 1.

**TABLE 1:** The function, shedding enzyme and protection strategies of glycocalyx in cerebrovascular.

(A) Functions	Regulation of vascular permeability	Mechanical barrier and charge barrier	Deen et al., 2001; Kutuzov et al., 2018; Zhu et al., 2018
	Regulation of vascular tone	Inducing and transmitting the shear stress change signal to the endothelial cells to synthesize and release nitric oxide	Ebong et al., 2014; Yen et al., 2015; Bartosch et al., 2017
	Attenuation of leukocyte adhesion	Reducing leukocyte contact with ICAM-1,ICAM-2, and VCAM-1	Van den Berg et al., 2006; Kim et al., 2013
	Attenuation of platelet adhesion	Reducing platelet contact with PECAM-1	Bell et al., 2017; Lupu et al., 2020
(B) Major shedding enzyme	MMPs	Cleaving core protein backbone of glycocalyx, directly	Endo et al., 2003; Song et al., 2015; Reine et al., 2019; Ali et al., 2019
	HPSE	Cutting HS	Shteper et al., 2003; Baraz et al., 2006; Qu et al., 2016; Zheng et al., 2016
	HAase	Cutting HA	Nieuwdorp et al., 2007; Becker et al., 2015
(C) Protection strategies of glycocalyx	Glucocorticoid	Stabilizing mast cells	Cui et al., 2015; Yu et al., 2019
	Antithrombin agents	Stabilizing glycocalyx structure by combining with it	Chappell et al., 2009a,b
	Abumin	Similar to that of antithrombin	Becker et al., 2015; Aldecoa et al., 2020
	Etanercept	TNF-α inhibitor	Nieuwdorp et al., 2009
	Sulodexide	Inhibiting HPSE and MMPs activities	Mannello and Raffetto, 2011; van Haare et al., 2017
	Doxycycline and batimastat	Inhibitors of MMPs	Lipowsky et al., 2011; Lipowsky and Lescanic, 2013
	Sevoflurane	Reduce MMPs production	Annecke et al., 2010; Fang et al., 2021

*ICAM, intercellular adhesion molecules; VCAM, vascular cell adhesion molecule; PECAM, platelet/endothelial cell adhesion molecule; HPSE, heparinase; HAase, hyaluronidase; MMPs, matrix metalloproteinases; TIMPs tissue inhibitor of matrix metalloproteinases; HDAC, histone deacetylase; HA, hyaluronic acid; HS, heparan sulfate.*

### Major Shedding Enzyme Responsible for Glycocalyx Damage

**FIGURE 3:**
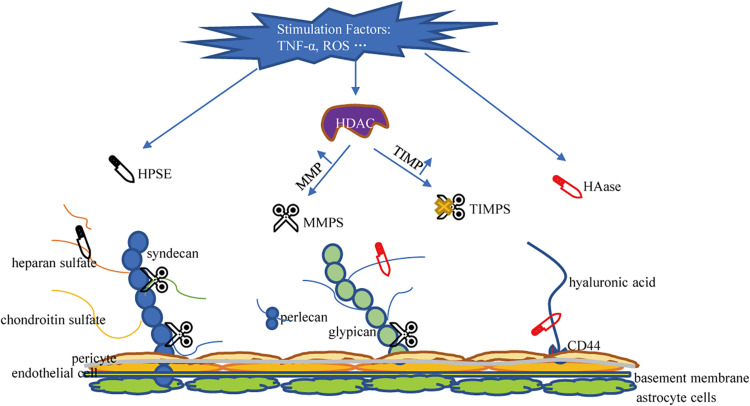
Mechanisms of glycocalyx damage. HPSE, Heparanase; HAase, hyaluronidase; MMPS, matrix metalloproteinases; TIMPS, tissue inhibitor of matrix metalloproteinases; HDAC, histone deacetylase; HA, hyaluronic acid; HS, heparan sulfate; CS, chondroitin sulfate.

The glycocalyx is degraded via inflammatory mechanisms such as MMPs, HPSE, and HAase. These sheddases are activated by reactive oxygen species and pro-inflammatory cytokines such as tumor necrosis factor alpha and interleukin-1beta (Iba and Levy, 2019; Uchimido et al., 2019). Several studies have determined that MMPs is the primary molecule responsible for glycocalyx degradation (Song et al., 2015). MMP-2, MMP-7, and MMP-9 directly cleave CS, MMP-1 cleaves syndecan-1, and MMP-9 is the main shedding enzyme of syndecan-4 (Endo et al., 2003; Reine et al., 2019). ADAM17 also participates in glycocalyx degradation by removing the extracellular domain of syndecan-4 (Piperigkou et al., 2016). In addition, studies have confirmed that ADAM15 causes vascular BBB dysfunction by inducing glycocalyx degradation. The underlying mechanism includes ADAM15-mediated CD44 cleavage and the release of the extracellular domain (HA) into the circulation, thereby promoting hyperpermeability of blood vessels and BBB destruction (Yang et al., 2018). Therefore, blocking ADAM15 may be a potential strategy to maintain the integrity of the glycocalyx. MMP is regulated by the activity of histone deacetylase (HDAC) inhibitors. When HDAC is up-regulated under stimulation, the expression of tissue inhibitors of matrix metalloproteinases (TIMPs) decreases and the expression of MMP increases, leading to accelerated glycocalyx degradation in endothelial cells (Ali et al., 2019). Ischemia and hypoxia can induce the activation of mast cells, so that the HPSE stored in the mast cells is released into the extracellular space, resulting in cleavage of HS from the endothelial glycocalyx (Becker et al., 2010b). HPSE is the only enzyme known to cleave HS and is another important factor that promotes the shedding of the glycocalyx (Becker et al., 2015). Research on HPSE has helped elucidate the catabolic processes involved in the decomposition of HS. Methylation of the HPSE promoter may regulate HPSE expression (Shteper et al., 2003). Recently, the transcription factor SMAD4, a key protein in the TGF-β signaling pathway, was found to inhibit HPSE by binding to the HPSE promoter region (Qu et al., 2016; Zheng et al., 2016). The inhibitory effect of p53 combined with the promoter on HPSE expression also resulted in the decrease of HPSE activity, indicating p53 is an effective regulator of HPSE expression (Baraz et al., 2006). Enzyme that promotes the shedding of HA is HAase. HAase cracks HA. Atherosclerosis and HAase activity is related (Nieuwdorp et al., 2007). Volume overload is encountered during neurosurgery. Volume overload will cause an increase in the release of natriuretic peptides. Experiments showed that A-, B-, and C-type natriuretic peptides have the ability to promote glycocalyx shedding (Jacob et al., 2013). A summary of the mechanism of damage to glycocalyx shedding is shown in Figure 3.

### Potential Strategies of Clinical Protection

The physiological function of the BBB is inseparable from the complete glycocalyx structure. The search for measures to protect the glycocalyx from degradation has always been a research hotspot. Glucocorticoid can stabilize mast cells, inhibit the activation of white blood cells, relieve the downstream inflammatory response, and protect glycocalyx, but its clinical application is limited by the adverse complications of immunosuppression caused by large doses (Cui et al., 2015; Yu et al., 2019). Antithrombin agents can stabilize its structure by combining with endothelial glycocalyx, thereby reducing the enzymatic decomposition of glycocalyx. However, the use of antithrombin during neurosurgery will affect the coagulation function of patients and cause adverse events of postoperative massive bleeding (Chappell et al., 2009a,b). The protective mechanism of albumin is similar to that of antithrombin, but excessive use of albumin will increase the cost of hospitalization for patients, and albumin is an allogeneic source, which will increase the risk of allergy in patients (Becker et al., 2015; Aldecoa et al., 2020). TNF-α inhibitor etanercept has been reported to have a protective effect, but the effect needs to be further studied (Nieuwdorp et al., 2009). Sulodexide has anti-inflammatory, anticoagulant and vascular protection effects, which may be achieved by inhibiting HPSE and MMP activities to reduce glycocalyx shedding (Mannello and Raffetto, 2011; van Haare et al., 2017). Doxycycline and batimastat, all rather non-selective inhibitors of MMPs, can attenuate syndecan and glycan shedding (Lipowsky et al., 2011; Lipowsky and Lescanic, 2013). In addition, sevoflurane has been shown to have a certain protective effect on the glycocalyx. The application of sevoflurane anesthesia in neurosurgery may be more beneficial to the protection of the BBB function (Annecke et al., 2010; Fang et al., 2021).

## Conclusion and Future Directions of Research

Vascular endothelial glycocalyx plays an indispensable role in BBB, such as inflammation, vascular permeability, blood coagulation, and vascular tone. However, it is not clear whether the glycocalyx in the BBB is different from the glycocalyx in the general vascular structure. Reviewing the relevant literature, details on the neuro-specific contributions of the glycocalyx are still lacking. In addition, the structural and functional relationships between glycocalyx and pericytes are also worth exploring. The therapeutic strategies for glycocalyx also need further research because the drugs reported in the current research will inevitably have some adverse reactions or application limitations. Therefore, innovative strategies in this emerging field of experimental medicine are desperately needed.

## Author Contributions

JC and XW were involved in the study design. WG, HC, and JW provided and prepared the samples. JJ and FF wrote the manuscript. All authors contributed to the article and approved the submitted version.

## Conflict of Interest

The authors declare that the research was conducted in the absence of any commercial or financial relationships that could be construed as a potential conflict of interest.

## Publisher’s Note

All claims expressed in this article are solely those of the authors and do not necessarily represent those of their affiliated organizations, or those of the publisher, the editors and the reviewers. Any product that may be evaluated in this article, or claim that may be made by its manufacturer, is not guaranteed or endorsed by the publisher.

## References

[B1] Abdullahi W. Tripathi D. Ronaldson P. T. (2018). Blood-brain barrier dysfunction in ischemic stroke: targeting tight junctions and transporters for vascular protection. *Am. J. Physiol. Cell Physiol.* 315 C343–C356. 10.1152/ajpcell.00095.2018 29949404 PMC6171039

[B2] Abrahamson E. E. Ikonomovic M. D. (2020). Brain injury-induced dysfunction of the blood brain barrier as a risk for dementia. *Exp. Neurol.* 328:113257. 10.1016/j.expneurol.2020.113257 32092298

[B3] Aldecoa C. Llau J. V. Nuvials X. Artigas A. (2020). Role of albumin in the preservation of endothelial glycocalyx integrity and the microcirculation: a review. *Ann. Intensive Care* 10:85. 10.1186/s13613-020-00697-1 32572647 PMC7310051

[B4] Ali M. M. Mahmoud A. M. Le Master E. Levitan I. Phillips S. A. (2019). Role of matrix metalloproteinases and histone deacetylase in oxidative stress-induced degradation of the endothelial glycocalyx. *Am. J. Physiol. Heart Circ. Physiol.* 316 H647–H663. 10.1152/ajpheart.00090.2018 30632766 PMC6459309

[B5] Alphonsus C. S. Rodseth R. N. (2014). The endothelial glycocalyx: a review of the vascular barrier. *Anaesthesia* 69 777–784. 10.1111/anae.12661 24773303

[B6] Annecke T. Chappell D. Chen C. Jacob M. Welsch U. Sommerhoff C. P. (2010). Sevoflurane preserves the endothelial glycocalyx against ischaemia-reperfusion injury. *Br. J. Anaesth.* 104 414–421. 10.1093/bja/aeq019 20172938

[B7] Araibi H. van der Merwe E. Gwanyanya A. Kelly-Laubscher R. (2020). The effect of sphingosine-1-phosphate on the endothelial glycocalyx during ischemia-reperfusion injury in the isolated rat heart. *Microcirculation* 27:e12612. 10.1111/micc.12612 32017300

[B8] Baraz L. Haupt Y. Elkin M. Peretz T. Vlodavsky I. (2006). Tumor suppressor p53 regulates heparanase gene expression. *Oncogene* 25 3939–3947. 10.1038/sj.onc.1209425 16474844

[B9] Bartosch A. M. W. Mathews R. Tarbell J. M. (2017). Endothelial Glycocalyx-Mediated Nitric Oxide Production in Response to Selective AFM Pulling. *Biophys. J.* 113 101–108. 10.1016/j.bpj.2017.05.033 28700908 PMC5510764

[B10] Becker B. F. Chappell D. Jacob M. (2010b). Endothelial glycocalyx and coronary vascular permeability: the fringe benefit. *Basic Res. Cardiol.* 105 687–701. 10.1007/s00395-010-0118-z 20859744

[B11] Becker B. F. Chappell D. Bruegger D. Annecke T. Jacob M. (2010a). Therapeutic strategies targeting the endothelial glycocalyx: acute deficits, but great potential. *Cardiovasc. Res.* 87 300–310. 10.1093/cvr/cvq137 20462866

[B12] Becker B. F. Jacob M. Leipert S. Salmon A. H. Chappell D. (2015). Degradation of the endothelial glycocalyx in clinical settings: searching for the sheddases. *Br. J. Clin. Pharmacol.* 80 389–402. 10.1111/bcp.12629 25778676 PMC4574825

[B13] Bell J. D. Rhind S. G. Di Battista A. P. Macdonald R. L. Baker A. J. (2017). Biomarkers of Glycocalyx Injury are Associated with Delayed Cerebral Ischemia Following Aneurysmal Subarachnoid Hemorrhage: a Case Series Supporting a New Hypothesis. *Neurocrit. Care* 26 339–347. 10.1007/s12028-016-0357-4 28000132

[B14] Bernfield M. Gotte M. Park P. W. Reizes O. Fitzgerald M. L. Lincecum J. (1999). Functions of cell surface heparan sulfate proteoglycans. *Annu. Rev. Biochem.* 68 729–777. 10.1146/annurev.biochem.68.1.729 10872465

[B15] Bombeli T. Schwartz B. R. Harlan J. M. (1998). Adhesion of activated platelets to endothelial cells: evidence for a GPIIbIIIa-dependent bridging mechanism and novel roles for endothelial intercellular adhesion molecule 1 (ICAM-1), alphavbeta3 integrin, and GPIbalpha. *J. Exp. Med.* 187 329–339. 10.1084/jem.187.3.329 9449713 PMC2212123

[B16] Brettner F. von Dossow V. Chappell D. (2017). The endothelial glycocalyx and perioperative lung injury. *Curr. Opin. Anaesthesiol.* 30 36–41. 10.1097/ACO.0000000000000434 27941354

[B17] Butler M. J. Down C. J. Foster R. R. Satchell S. C. (2020). The Pathological Relevance of Increased Endothelial Glycocalyx Permeability. *Am. J. Pathol.* 190 742–751. 10.1016/j.ajpath.2019.11.015 32035881 PMC7163249

[B18] Chappell D. Hofmann-Kiefer K. Jacob M. Rehm M. Briegel J. Welsch U. (2009a). TNF-alpha induced shedding of the endothelial glycocalyx is prevented by hydrocortisone and antithrombin. *Basic Res. Cardiol.* 104 78–89. 10.1007/s00395-008-0749-5 18836678

[B19] Chappell D. Jacob M. Hofmann-Kiefer K. Rehm M. Welsch U. Conzen P. (2009b). Antithrombin reduces shedding of the endothelial glycocalyx following ischaemia/reperfusion. *Cardiovasc. Res.* 83 388–396. 10.1093/cvr/cvp097 19307232

[B20] Cui N. Wang H. Long Y. Su L. Liu D. (2015). Dexamethasone Suppressed LPS-Induced Matrix Metalloproteinase and Its Effect on Endothelial Glycocalyx Shedding. *Mediators Inflamm.* 2015:912726. 10.1155/2015/912726 26199464 PMC4493300

[B21] Deen W. M. Lazzara M. J. Myers B. D. (2001). Structural determinants of glomerular permeability. *Am. J. Physiol. Renal Physiol.* 281 F579–F596. 10.1152/ajprenal.2001.281.4.F579 11553505

[B22] Devaraj S. Yun J. M. Adamson G. Galvez J. Jialal I. (2009). C-reactive protein impairs the endothelial glycocalyx resulting in endothelial dysfunction. *Cardiovasc. Res.* 84 479–484. 10.1093/cvr/cvp249 19620133 PMC2777951

[B23] Ebong E. E. Lopez-Quintero S. V. Rizzo V. Spray D. C. Tarbell J. M. (2014). Shear-induced endothelial NOS activation and remodeling via heparan sulfate, glypican-1, and syndecan-1. *Integr. Biol.* 6 338–347. 10.1039/c3ib40199e 24480876 PMC3996848

[B24] Ebong E. E. Macaluso F. P. Spray D. C. Tarbell J. M. (2011). Imaging the endothelial glycocalyx in vitro by rapid freezing/freeze substitution transmission electron microscopy. *Arterioscler. Thromb. Vasc. Biol.* 31 1908–1915. 10.1161/ATVBAHA.111.225268 21474821 PMC3141106

[B25] Endo K. Takino T. Miyamori H. Kinsen H. Yoshizaki T. Furukawa M. (2003). Cleavage of syndecan-1 by membrane type matrix metalloproteinase-1 stimulates cell migration. *J. Biol. Chem.* 278 40764–40770. 10.1074/jbc.M306736200 12904296

[B26] Fang F. Q. Sun J. H. Wu Q. L. Feng L. Y. Fan Y. X. Ye J. X. (2021). Protective effect of sevoflurane on vascular endothelial glycocalyx in patients undergoing heart valve surgery: a randomised controlled trial. *Eur. J. Anaesthesiol.* 38 477–486. 10.1097/EJA.0000000000001429 33399382

[B27] Hoogewerf A. J. Kuschert G. S. Proudfoot A. E. Borlat F. Clark-Lewis I. Power C. A. (1997). Glycosaminoglycans mediate cell surface oligomerization of chemokines. *Biochemistry* 36 13570–13578. 10.1021/bi971125s 9354625

[B28] Iba T. Levy J. H. (2019). Derangement of the endothelial glycocalyx in sepsis. *J. Thromb. Haemost.* 17 283–294. 10.1111/jth.14371 30582882

[B29] Jacob M. Saller T. Chappell D. Rehm M. Welsch U. Becker B. F. (2013). Physiological levels of A-, B- and C-type natriuretic peptide shed the endothelial glycocalyx and enhance vascular permeability. *Basic Res. Cardiol.* 108:347. 10.1007/s00395-013-0347-z 23563917

[B30] Kabedev A. Lobaskin V. (2018). Structure and elasticity of bush and brush-like models of the endothelial glycocalyx. *Sci. Rep.* 8:240. 10.1038/s41598-017-18577-3 29321567 PMC5762753

[B31] Kang H. Yang J. Zhang W. Lu J. Ma X. Sun A. (2021). Effect of endothelial glycocalyx on water and LDL transport through the rat abdominal aorta. *Am. J. Physiol. Heart Circ. Physiol.* 320 H1724–H1737. 10.1152/ajpheart.00861.2020 33710913

[B32] Kim D. H. Chung J. H. Yoon J. S. Ha Y. M. Bae S. Lee E. K. (2013). Ginsenoside Rd inhibits the expressions of iNOS and COX-2 by suppressing NF-kappaB in LPS-stimulated RAW264.7 cells and mouse liver. *J Ginseng Res.* 37 54–63. 10.5142/jgr.2013.37.54 23717157 PMC3659628

[B33] Koenig A. Norgard-Sumnicht K. Linhardt R. Varki A. (1998). Differential interactions of heparin and heparan sulfate glycosaminoglycans with the selectins. Implications for the use of unfractionated and low molecular weight heparins as therapeutic agents. *J. Clin. Invest.* 101 877–889. 10.1172/JCI1509 9466983 PMC508636

[B34] Kolarova H. Vitecek J. Cerna A. Cernik M. Pribyl J. Skladal P. (2021). Myeloperoxidase mediated alteration of endothelial function is dependent on its cationic charge. *Free Radic. Biol. Med.* 162 14–26. 10.1016/j.freeradbiomed.2020.11.008 33271281

[B35] Koo A. Dewey C. F. Jr. Garcia-Cardena G. (2013). Hemodynamic shear stress characteristic of atherosclerosis-resistant regions promotes glycocalyx formation in cultured endothelial cells. *Am. J. Physiol. Cell Physiol.* 304 C137–46. 10.1152/ajpcell.00187.2012 23114962 PMC3546807

[B36] Kozar R. A. Pati S. (2015). Syndecan-1 restitution by plasma after hemorrhagic shock. *J. Trauma Acute Care Surg.* 78 S83–S86. 10.1097/TA.0000000000000631 26002270 PMC4841450

[B37] Kutuzov N. Flyvbjerg H. Lauritzen M. (2018). Contributions of the glycocalyx, endothelium, and extravascular compartment to the blood-brain barrier. *Proc. Natl. Acad. Sci. U. S. A.* 115 E9429–E9438. 10.1073/pnas.1802155115 30217895 PMC6176561

[B38] Lipowsky H. H. Lescanic A. (2013). The effect of doxycycline on shedding of the glycocalyx due to reactive oxygen species. *Microvasc. Res.* 90 80–85. 10.1016/j.mvr.2013.07.004 23899417 PMC3852187

[B39] Lipowsky H. H. Sah R. Lescanic A. (2011). Relative roles of doxycycline and cation chelation in endothelial glycan shedding and adhesion of leukocytes. *Am. J. Physiol. Heart Circ. Physiol.* 300 H415–H422. 10.1152/ajpheart.00923.2010 21148759 PMC3044056

[B40] Lupu F. Kinasewitz G. Dormer K. (2020). The role of endothelial shear stress on haemodynamics, inflammation, coagulation and glycocalyx during sepsis. *J. Cell. Mol. Med.* 24 12258–12271. 10.1111/jcmm.15895 32951280 PMC7687012

[B41] Lyu N. Du Z. Qiu H. Gao P. Yao Q. Xiong K. (2020). Mimicking the Nitric Oxide-Releasing and Glycocalyx Functions of Endothelium on Vascular Stent Surfaces. *Adv Sci.* 7:2002330. 10.1002/advs.202002330 33173746 PMC7610264

[B42] Mannello F. Raffetto J. D. (2011). Matrix metalloproteinase activity and glycosaminoglycans in chronic venous disease: the linkage among cell biology, pathology and translational research. *Am. J. Transl. Res.* 3 149–158. 21416057 PMC3056561

[B43] Mende M. Bednarek C. Wawryszyn M. Sauter P. Biskup M. B. Schepers U. (2016). Chemical Synthesis of Glycosaminoglycans. *Chem. Rev.* 116 8193–8255. 10.1021/acs.chemrev.6b00010 27410264

[B44] Michel C. C. (1997). Starling: the formulation of his hypothesis of microvascular fluid exchange and its significance after 100 years. *Exp. Physiol.* 82 1–30. 10.1113/expphysiol.1997.sp004000 9023503

[B45] Mulivor A. W. Lipowsky H. H. (2004). Inflammation- and ischemia-induced shedding of venular glycocalyx. *Am. J. Physiol. Heart Circ. Physiol.* 286 H1672–H1680. 10.1152/ajpheart.00832.2003 14704229

[B46] Mulivor A. W. Lipowsky H. H. (2009). Inhibition of glycan shedding and leukocyte-endothelial adhesion in postcapillary venules by suppression of matrixmetalloprotease activity with doxycycline. *Microcirculation* 16 657–666. 10.3109/10739680903133714 19905966

[B47] Nandi A. Estess P. Siegelman M. H. (2000). Hyaluronan anchoring and regulation on the surface of vascular endothelial cells is mediated through the functionally active form of CD44. *J. Biol. Chem.* 275 14939–14948. 10.1074/jbc.275.20.14939 10809739

[B48] Nieuwdorp M. Holleman F. de Groot E. Vink H. Gort J. Kontush A. (2007). Perturbation of hyaluronan metabolism predisposes patients with type 1 diabetes mellitus to atherosclerosis. *Diabetologia* 50 1288–1293. 10.1007/s00125-007-0666-4 17415544 PMC1914278

[B49] Nieuwdorp M. Meuwese M. C. Mooij H. L. van Lieshout M. H. Hayden A. Levi M. (2009). Tumor necrosis factor-alpha inhibition protects against endotoxin-induced endothelial glycocalyx perturbation. *Atherosclerosis* 202 296–303. 10.1016/j.atherosclerosis.2008.03.024 18550063

[B50] Nikmanesh M. Cancel L. M. Shi Z. D. Tarbell J. M. (2019). Heparan sulfate proteoglycan, integrin, and syndecan-4 are mechanosensors mediating cyclic strain-modulated endothelial gene expression in mouse embryonic stem cell-derived endothelial cells. *Biotechnol. Bioeng.* 116 2730–2741. 10.1002/bit.27104 31282995

[B51] Piperigkou Z. Mohr B. Karamanos N. Gotte M. (2016). Shed proteoglycans in tumor stroma. *Cell Tissue Res.* 365 643–655. 10.1007/s00441-016-2452-4 27365088

[B52] Pries A. R. Secomb T. W. Gaehtgens P. (2000). The endothelial surface layer. *Pflugers Arch.* 440 653–666. 10.1007/s004240000307 11007304

[B53] Purcell S. C. Godula K. (2019). Synthetic glycoscapes: addressing the structural and functional complexity of the glycocalyx. *Interface Focus* 9:20180080. 10.1098/rsfs.2018.0080 30842878 PMC6388016

[B54] Qu H. Zheng L. Jiao W. Mei H. Li D. Song H. (2016). Smad4 suppresses the tumorigenesis and aggressiveness of neuroblastoma through repressing the expression of heparanase. *Sci. Rep.* 6:32628. 10.1038/srep32628 27595937 PMC5011643

[B55] Rabelink T. J. van den Berg B. M. Garsen M. Wang G. Elkin M. van der Vlag J. (2017). Heparanase: roles in cell survival, extracellular matrix remodelling and the development of kidney disease. *Nat. Rev. Nephrol.* 13 201–212. 10.1038/nrneph.2017.6 28163306

[B56] Rambourg A. Neutra M. Leblond C. P. (1966). Presence of a “cell coat” rich in carbohydrate at the surface of cells in the rat. *Anat. Rec.* 154 41–71. 10.1002/ar.1091540105 4162458

[B57] Rana A. Musto A. E. (2018). The role of inflammation in the development of epilepsy. *J. Neuroinflammation* 15:144. 10.1186/s12974-018-1192-7 29764485 PMC5952578

[B58] Rapraeger A. Jalkanen M. Endo E. Koda J. Bernfield M. (1985). The cell surface proteoglycan from mouse mammary epithelial cells bears chondroitin sulfate and heparan sulfate glycosaminoglycans. *J. Biol. Chem.* 260 11046–11052. 3161889

[B59] Reine T. M. Lanzalaco F. Kristiansen O. Enget A. R. Satchell S. Jenssen T. G. (2019). Matrix metalloproteinase-9 mediated shedding of syndecan-4 in glomerular endothelial cells. *Microcirculation* e12534. 10.1111/micc.12534 [Online ahead of print]. 30703289

[B60] Reitsma S. Slaaf D. W. Vink H. van Zandvoort M. A. Oude Egbrink M. G. (2007). The endothelial glycocalyx: composition, functions, and visualization. *Pflugers Arch.* 454 345–359. 10.1007/s00424-007-0212-8 17256154 PMC1915585

[B61] Ruegg C. Mariotti A. (2003). Vascular integrins: pleiotropic adhesion and signaling molecules in vascular homeostasis and angiogenesis. *Cell. Mol. Life Sci.* 60 1135–1157. 10.1007/s00018-003-2297-3 12861381 PMC11138931

[B62] Salmon A. H. Satchell S. C. (2012). Endothelial glycocalyx dysfunction in disease: albuminuria and increased microvascular permeability. *J. Pathol.* 226 562–574. 10.1002/path.3964 22102407

[B63] Santa-Maria A. R. Walter F. R. Figueiredo R. Kincses A. Vigh J. P. Heymans M. (2021). Flow induces barrier and glycocalyx-related genes and negative surface charge in a lab-on-a-chip human blood-brain barrier model. *J. Cereb. Blood Flow Metab.* 41 2201–2215. 10.1177/0271678X21992638 33563079 PMC8393308

[B64] Schmidt E. P. Yang Y. Janssen W. J. Gandjeva A. Perez M. J. Barthel L. (2012). The pulmonary endothelial glycocalyx regulates neutrophil adhesion and lung injury during experimental sepsis. *Nat. Med.* 18 1217–1223. 10.1038/nm.2843 22820644 PMC3723751

[B65] Schnoor M. Alcaide P. Voisin M. B. van Buul J. D. (2015). Crossing the Vascular Wall: common and Unique Mechanisms Exploited by Different Leukocyte Subsets during Extravasation. *Mediators Inflamm.* 2015:946509. 10.1155/2015/946509 26568666 PMC4629053

[B66] Shteper P. J. Zcharia E. Ashhab Y. Peretz T. Vlodavsky I. Ben-Yehuda D. (2003). Role of promoter methylation in regulation of the mammalian heparanase gene. *Oncogene* 22 7737–7749. 10.1038/sj.onc.1207056 14586400

[B67] Simard J. M. Tosun C. Ivanova S. Kurland D. B. Hong C. Radecki L. (2012). Heparin reduces neuroinflammation and transsynaptic neuronal apoptosis in a model of subarachnoid hemorrhage. *Transl. Stroke Res.* 3 155–165. 10.1007/s12975-012-0166-9 22707992 PMC3372778

[B68] Song J. Wu C. Korpos E. Zhang X. Agrawal S. M. Wang Y. (2015). Focal MMP-2 and MMP-9 activity at the blood-brain barrier promotes chemokine-induced leukocyte migration. *Cell Rep.* 10 1040–1054. 10.1016/j.celrep.2015.01.037 25704809

[B69] Sperandio M. (2006). Selectins and glycosyltransferases in leukocyte rolling in vivo. *FEBS J.* 273 4377–4389. 10.1111/j.1742-4658.2006.05437.x 16956372

[B70] Starling E. H. (1896). On the Absorption of Fluids from the Connective Tissue Spaces. *J. Physiol.* 19 312–326. 10.1113/jphysiol.1896.sp000596 16992325 PMC1512609

[B71] Tarbell J. M. (2010). Shear stress and the endothelial transport barrier. *Cardiovasc. Res.* 87 320–330. 10.1093/cvr/cvq146 20543206 PMC2915475

[B72] Uchimido R. Schmidt E. P. Shapiro N. I. (2019). The glycocalyx: a novel diagnostic and therapeutic target in sepsis. *Crit. Care* 23:16. 10.1186/s13054-018-2292-6 30654825 PMC6337861

[B73] Ushiyama A. Kataoka H. Iijima T. (2016). Glycocalyx and its involvement in clinical pathophysiologies. *J. Intensive Care* 4:59. 10.1186/s40560-016-0182-z 27617097 PMC5017018

[B74] Van den Berg B. M. Nieuwdorp M. Stroes E. S. Vink H. (2006). Glycocalyx and endothelial (dys) function: from mice to men. *Pharmacol. Rep.* 58 75–80. 17332675

[B75] van Golen R. F. Reiniers M. J. Vrisekoop N. Zuurbier C. J. Olthof P. B. van Rheenen J. (2014). The Mechanisms and Physiological Relevance of Glycocalyx Degradation in Hepatic Ischemia/Reperfusion Injury. *Antioxid. Redox Sign.* 21:1098. 10.1089/ars.2013.5751 24313895 PMC4123469

[B76] van Haare J. Kooi M. E. van Teeffelen J. W. Vink H. Slenter J. Cobelens H. (2017). Metformin and sulodexide restore cardiac microvascular perfusion capacity in diet-induced obese rats. *Cardiovasc. Diabetol.* 16:47. 10.1186/s12933-017-0525-7 28399917 PMC5387275

[B77] van Haaren P. M. VanBavel E. Vink H. Spaan J. A. (2003). Localization of the permeability barrier to solutes in isolated arteries by confocal microscopy. *Am. J. Physiol. Heart Circ. Physiol.* 285 H2848–56. 10.1152/ajpheart.00117.2003 12907418

[B78] Yang X. Meegan J. E. Jannaway M. Coleman D. C. Yuan S. Y. (2018). A disintegrin and metalloproteinase 15-mediated glycocalyx shedding contributes to vascular leakage during inflammation. *Cardiovasc. Res.* 114 1752–1763. 10.1093/cvr/cvy167 29939250 PMC6198742

[B79] Yen W. Cai B. Yang J. Zhang L. Zeng M. Tarbell J. M. (2015). Endothelial surface glycocalyx can regulate flow-induced nitric oxide production in microvessels in vivo. *PLoS One* 10:e0117133. 10.1371/journal.pone.0117133 25575016 PMC4289188

[B80] Yu W. Q. Zhang S. Y. Fu S. Q. Fu Q. H. Lu W. N. Zhang J. (2019). Dexamethasone protects the glycocalyx on the kidney microvascular endothelium during severe acute pancreatitis. *J. Zhejiang Univ. Sci. B* 20 355–362. 10.1631/jzus.B1900006 30932380 PMC6454317

[B81] Zenaro E. Piacentino G. Constantin G. (2017). The blood-brain barrier in Alzheimer’s disease. *Neurobiol. Dis.* 107 41–56. 10.1016/j.nbd.2016.07.007 27425887 PMC5600438

[B82] Zhao F. Zhong L. Luo Y. (2021). Endothelial glycocalyx as an important factor in composition of blood-brain barrier. *CNS Neurosci. Ther.* 27 26–35. 10.1111/cns.13560 33377610 PMC7804892

[B83] Zheng L. Jiao W. Song H. Qu H. Li D. Mei H. (2016). miRNA-558 promotes gastric cancer progression through attenuating Smad4-mediated repression of heparanase expression. *Cell Death Dis.* 7:e2382. 10.1038/cddis.2016.293 27685626 PMC5059886

[B84] Zhu J. Li X. Yin J. Hu Y. Gu Y. Pan S. (2017). Glycocalyx degradation leads to blood-brain barrier dysfunction and brain edema after asphyxia cardiac arrest in rats. *J. Cereb. Blood Flow Metab.* 38 1979–1992. 10.1177/0271678X17726062 28825336 PMC6259325

[B85] Zhu J. Li X. Yin J. Hu Y. Gu Y. Pan S. (2018). Glycocalyx degradation leads to blood-brain barrier dysfunction and brain edema after asphyxia cardiac arrest in rats. *J. Cereb. Blood Flow Metab.* 38 1979–1992. 28825336 10.1177/0271678X17726062 PMC6259325

[B86] Zuurbier C. J. (2019). Ketamine-(Dex)Medetomidine, Hyperglycemia, Glycocalyx, and Vascular Permeability. *Anesth. Analg.* 129:e102. 10.1213/ANE.0000000000004181 31425237

